# Keystone Epitope Theory: Implications for Hypersensitivity, Autoimmunity, and Transplantation

**DOI:** 10.20411/pai.v11i2.1016

**Published:** 2026-08-07

**Authors:** Simon Mallal, Amir Asiaee, Elizabeth Phillips

**Affiliations:** 1 Department of Medicine, Vanderbilt University Medical Center, Nashville, Tennessee; 2 Institute for Immunology and Infectious Diseases, Murdoch University, Perth, Western Australia, Australia; 3 Department of Biostatistics, Vanderbilt University Medical Center, Nashville, Tennessee

**Keywords:** Drug Hypersensitivity, HLA, Peptide–HLA, TCR, Tissue-resident Memory, Heterologous Immunity, Modified Self, Keystone Epitope Theory, Herpesvirus

## Abstract

HLA class I alleles confer a striking risk for T cell–mediated drug hypersensitivity, yet positive predictive values are low—typically under 10% and as low as 0.12% for some drug–HLA pairs. We propose that persistent, human-adapted pathogens—notably herpesviruses—focus postnatal immune memory on conserved epitopes in the tissue niches where viral control occurs (the Keystone Epitope Theory). The phylogenetic basis for this proposal is that herpesviruses and their vertebrate hosts have co-adapted over hundreds of millions of years, and that this co-evolutionary relationship is replayed ontogenetically as each individual acquires these infections and builds tissue-specific immune memory. When a drug-altered self-peptide approximates the geometry of such a target and is presented by the same risk HLA in the same niche at sufficient density, pre-existing tissue-resident memory T cells (TRM) may be recruited, breaching local regulatory equilibria and driving immunopathology. We synthesize three strands of evidence: (i) heterologous immunity, in which virus-imprinted TRM cross-recognize drug-modified self; (ii) antigen presentation in the same tissue where antiviral memory already resides, which helps explain why injury is tissue-restricted; and (iii) public and private TCR solutions that bridge viral and self-targets. Beginning with T cell–mediated drug hypersensitivity as an empirical anchor, we extend this framework to EBV-associated multiple sclerosis and transplant rejection and conclude with proposed experimental validation strategies that may be applicable more broadly to T cell–mediated hypersensitivity and autoimmunity.

## Graphical abstract

**Figure:**
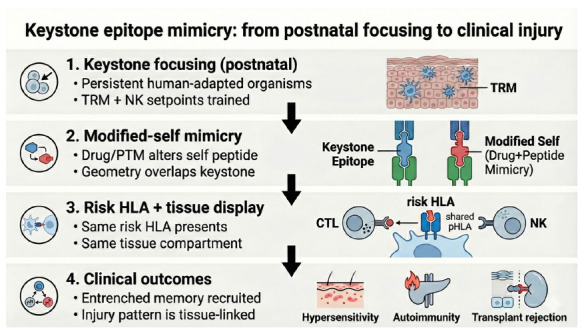
**From keystone protection to clinical misdirection.** Postnatal immune focusing concentrates durable tissue-resident memory on conserved peptide–HLA targets in specific niches. Modified self-peptides (drug-altered or post-translationally modified) that approximate this geometry, presented by the same risk HLA in the same tissue, can recruit entrenched antiviral programs and, when mimicking ligand burden is sufficient, exceed local inhibitory set-points. The clinical result is high NPV but low PPV for HLA risk alleles. Abbreviations: TRM, tissue-resident memory T cell; PTM, post-translational modification.

## INTRODUCTION

HLA class I associations with T cell–mediated drug hypersensitivity rank among the strongest genetic risk factors in medicine, yet positive predictive values range from 0.12% (HLA-B*57:01 and flucloxacillin DILI) to approximately 55% (HLA-B*57:01 and abacavir hypersensitivity), with most HLA-drug pairs well under 10% [1, 2]. This paradox—high negative predictive value (NPV) but low positive predictive value (PPV)—implies that HLA is necessary but not sufficient and that additional conditions must be met before immunopathology occurs.

One explanatory model—the Keystone Epitope Theory—proposes that persistent, co-evolved human pathogens, including cytomegalovirus (CMV), Epstein-Barr virus (EBV), human herpes-virus 6 (HHV-6), herpes simplex virus types 1 (HSV-1) and -2, varicella-zoster virus (VZV), and possibly *Mycobacterium tuberculosis* [3], shape postnatal immune priorities by repeatedly stimulating conserved epitopes in the tissue niches where pathogen control must occur. In this model, persistent organisms in specific niches establish a dynamic equilibrium between effector and tolerizing responses [4]; pathology occurs when a drug-altered self-peptide of qualitatively appropriate geometry is presented at quantitatively sufficient density by the same risk HLA in the same tissue niche, exceeding a local regulatory threshold and recruiting tissue-positioned antiviral memory against self [5, 6]. Although keystone organisms are proposed to be the most specifically human-adapted apex chronic persistent organisms, other immune niche-calibrating organisms have also been identified [3].

This review uses T cell-mediated drug hypersensitivity as the primary empirical anchor, extends the framework briefly to EBV-associated multiple sclerosis and transplant rejection, and proposes experimental approaches for validation. Throughout, we distinguish established evidence from our theoretical model and its testable predictions.

## MECHANISTIC SUBSTRATE

### Postnatal Immune Focusing

**Figure 1.**
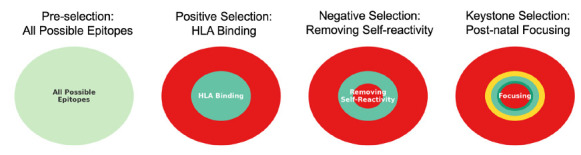
**Three-stage selection culminating in postnatal epitope focusing.** (A) Pre-selection: the universe of possible epitopes. (B) Positive selection: HLA binding restricts the immunopeptidome repertoire. (C) Negative selection: central tolerance removes high-affinity self-reactivity. (D) Postnatal focusing: repeated lifelong exposure to conserved, function-linked epitopes in their tissue niche concentrates tissue-resident memory on a narrower band of targets.

The immune system shapes its T-cell repertoire through thymic positive selection for HLA-restricted binding, followed by negative selection to eliminate high-affinity self-reactive clones. We propose that a third stage of immune training occurs after birth, driven by persistent, co-evolved pathogens (Figure 1) whose conserved epitopes focus immune memory on evolutionarily constrained targets [6]. A corollary is that modified self-peptides mimicking these epitopes may overcome local peripheral tolerance, potentially allowing pre-existing memory T cells to mount pathogenic responses.

The regulatory gates that restrain these responses are not uniform. Priming-stage control (for example, CTLA-4–dependent costimulatory modulation) shapes which clones enter durable competence states, whereas tissue-stage restraint—including PD-1–dependent gain modulation, local regulatory T cells, and other inhibitory circuits—adjusts effector output without eliminating competence [4, 7]. Checkpoint blockade preferentially perturbs the latter, lowering effector thresholds in tissues that already contain entrenched memory (Supplementary Figure S10.1).

### Tissue-resident Memory and Cross-reactive Vulnerability

Persistent, human-adapted DNA viruses (notably human herpesviruses) establish durable immune memory throughout life; they seed tissue-resident compartments, maintain recall through intermittent antigenic stimulation, and bias receptor selection toward constrained, function-linked epitopes. Population-scale sequencing has shown that persistent EBV DNA burden is a measurable, host regulated trait shaped by antigen-processing variation and MHC class II genotype [8], suggesting that what matters is not simply whether exposure occurred, but how persistent viral material is controlled, processed, and presented by the host immune system. Large-scale virome studies further report that herpesvirus persistence is associated with virus-specific MHC architecture, with ERAP1, ERAP2, and LNPEP—all peptide-trimming enzymes that influence which peptides become available for antigen presentation—shaping peptide availability in a virus-specific manner [9]. Blood and saliva show distinct host-genetic architectures for viral burden [9]. Independently, HLA-B*08:01 differentially associates with type 1 versus type 2 EBV, showing that host–virus genotype interactions can also shape persistence [9]. Together, these observations underscore that the genetic determinants of viral burden and the determinants of epitope-specific immune responses are related but not identical.

Marked variation in HLA alleles, killer cell immunoglobulin-like receptor (KIR) haplotypes, and other immunogenetic markers has been shaped in part by the selective pressures of locally co-adapted pathogens, including geographically structured herpesvirus polymorphisms [10, 11]. Modern migration and globalization can disconnect these evolved immunogenetic backgrounds from local pathogen variants, and changes in the timing of early-life viral acquisition may further alter immune calibration (Supplementary Figure S10.3). We emphasize that sanitation, vaccination, antibiotics, shelter, nutrition, and safer childbirth are overwhelming population-level benefits; any coevolutionary mismatch hypothesis concerns past evolutionary immune calibration, not a critique of modern public health.

A single peptide–HLA (pHLA) complex is read by two cytotoxic arms: CD8 T cells via the TCR and NK cells via KIR/CD94–NKG2. Evidence suggests that effector set-points are influenced by (i) inhibitory tone (notably HLA-C level), (ii) peptide/HLA stability and loading (tapasin/TAPBP, ERAP), and (iii) licensing and education history, which CMV/EBV exposure and ancestry shape over time [12]. The coordinated but independently tunable nature of CTL and NK pressure on a given tissue means that inhibitory NK tone can be preserved while CTL visibility falls (or vice versa), explaining why some drug-altered peptidomes trigger CTL without disinhibiting NK. KIR/HLA genotype may further modify the balance between CTL and NK effector thresholds.

**Figure 2.**
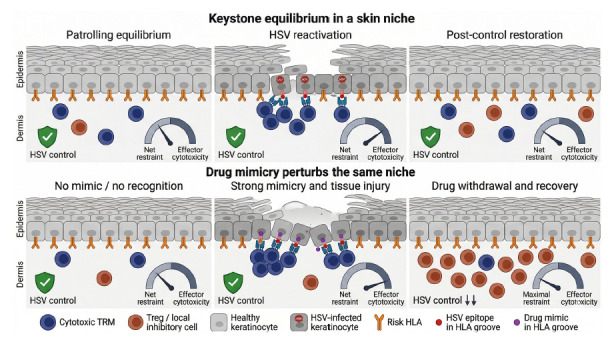
**Dynamic equilibrium between niche-resident antiviral memory and local restraint in skin, and its perturbation by drug mimicry.** Top row: The figure uses HSV, or by analogy VZV, in skin as an example of a niche-calibrating organism. In *Patrolling equilibrium*, sparse tissue-resident memory T cells patrol a skin niche in the presence of constitutive HLA class I expression, while local inhibitory circuits maintain restraint. In *HSV reactivation*, focal viral reactivation transiently shifts the niche toward effector cytotoxicity: infected keratinocytes display viral epitope in the same HLA context, tissue-resident cytotoxic T cells expand locally, and limited epithelial injury accompanies viral control without implying escape from control. In *post-control restoration*, once viral control is restored, inhibitory circuits expand, effector activity contracts, and tissue integrity recovers. Bottom row: The same niche is perturbed by drug mimicry. In *No mimic / no recognition*, drug exposure does not generate a sufficiently similar target, so pre-existing antiviral memory is not recruited and the niche remains quiet. In *Strong mimicry and tissue injury*, a rare structurally appropriate neoantigen displayed by the same risk HLA across many keratinocytes recruits pre-positioned antiviral memory and produces tissue injury when both qualitative similarity and quantitative target burden exceed local tolerizing capacity. In *Drug withdrawal and recovery*, withdrawal of the drug reduces neoantigen display and local tissue recovery begins, but the temporary shift toward restraint may also reduce local antiviral control and create a window of herpesvirus reactivation risk, a possibility relevant to the dynamics of DRESS. Although illustrated here in skin with HSV or VZV, the same logic may extend to other tissue niches, other niche-calibrating organisms, and other rare structural neoantigens capable of initiating cross-reactive pathology.

Persistent pathogens establish tissue-specific reservoirs and immune surveillance. HSV-specific CD8+ T cells persist at the dermal-epidermal junction [39], whereas CMV infects fibroblasts, epithelial cells, endothelial cells, smooth muscle cells, and monocytes [13, 14]. When modified self is displayed in one of these tissue compartments, entrenched cytotoxic programs can be recruited without requiring classical priming of a naive response and can trigger injury when local regulatory thresholds are exceeded (Figure 2). The same logic applies to alloreactivity, where virus-imprinted TRM can cross-recognize donor pHLA in transplanted tissue (see Supplementary Material S5).

In this framework, a niche should not be read simply as an organ label. Anatomical terms such as skin, lymph node, vascular endothelium, or reticuloendothelial tissue are useful shorthand, but the operative unit is the cell type and antigen-presentation circuit in which the relevant pHLA is displayed, and immune control is maintained.

**Figure 3.**
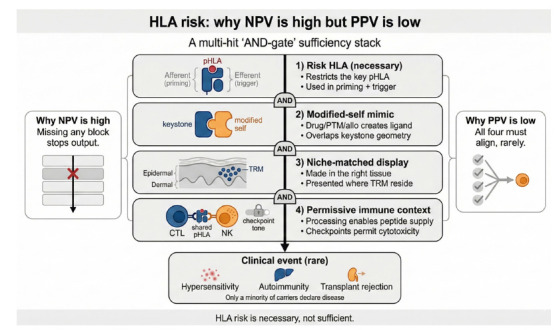
**Why risk HLA has high NPV but low PPV.** HLA risk is necessary but rarely sufficient. Clinical disease emerges only when multiple conditions co-occur: (i) risk HLA; (ii) a modified-self ligand that matches the geometry of an entrenched viral target and is present at sufficient burden to breach local inhibitory set-points; (iii) tissue-restricted generation and presentation in the niche where resident memory sits; and (iv) permissive checkpoint and regulatory context. This explains why screening can rule out risk but poorly predicts who will develop disease without additional biomarkers.

Unlike most modified self-antigens, which are actively tolerized [15, 16], persistent viral epitopes sustain durable immune effectors because ongoing surveillance is required for control of latent infection or reactivation. Persistent viral infection, herpesvirus evolution, and viral interference with host chemokine signaling are well documented [17–19]. Separately, herpesvirus-specific T cells can cross-recognize conserved or related peptides across different herpesviruses [20, 21]. Together, these observations provide a plausible, but not direct, basis for testing whether virus-trained T-cell responses can cross-recognize drug-modified self when the quality of the mimic geometry and the quantity of neoantigen targets exceed local niche tolerance capacity. These multiple, individually necessary conditions are summarized in Figure 3.

## T CELL–MEDIATED DRUG HYPERSENSITIVITY

### Abacavir vs Acyclovir: Repertoire Alteration is Not Sufficient

Comparative studies of abacavir and acyclovir, two drugs that modify the peptide repertoire presented by HLA-B*57:01, directly illustrate this vulnerability. Both drugs bind non-covalently to the peptide-binding groove and alter the preferences of anchor residues [22, 23]. Abacavir binds beneath the peptide within the HLA-B*57:01 groove, altering the self-peptide repertoire [22]. This generates a large number of novel peptide–HLA complexes perceived as foreign by CD8+ T cells, yet approximately 45% of HLA-B*57:01 carriers exposed to abacavir remain clinically tolerant [1]; mouse models have shown that this tolerance is actively maintained by CD4+ T cells [16, 24]. Acyclovir induces a far more modest repertoire shift and is not associated with hypersensitivity [23], underscoring that repertoire alteration alone is insufficient—only certain altered repertoires, those that structurally approximate targets of entrenched viral memory, are capable of mobilizing effector responses. Carbamazepine does not bind to the floor of the HLA groove as abacavir does. Rather, carbamazepine-associated SJS/TEN appears to involve direct drug-dependent recognition of peptide-loaded HLA-B*15:02 [25]. Clinical disease requires a rare convergence: a drug-dependent change in pHLA that introduces structural motifs cross-recognized by virus-trained TCRs, in the relevant tissue niche, and in a local context where regulatory tone permits the composite signal to breach the local inhibitory threshold.

Abacavir is the clearest example of drug-altered HLA-B*57:01 self-presentation. Structural and immunopeptidomic studies show that abacavir binds within the HLA-B*57:01 groove, changes F-pocket chemistry, and shifts the presented repertoire toward peptides with small hydrophobic C-terminal residues, particularly valine and isoleucine, rather than the large aromatic residues normally favored by HLA-B*57:01 [22]. T cells from abacavir-hypersensitive donors can recognize abacavir-dependent self-peptide-HLA complexes, including VTTDIQVKV in the presence of abacavir [22]. However, no viral or microbial epitope has yet been definitively shown to be the initiating cross-reactive epitope in clinical abacavir hypersensitivity.

Sooda et al provide an important roadmap and an equally important boundary condition [26]. They mapped HLA-B*57:01-restricted EBV responses, identified EBV epitopes including EBNA3C QSRGDENRGW and EBNA3B VSFIEFVGW, and cloned dominant EBV-specific TCRs. These TCRs retained cognate EBV specificity but did not cross-recognize abacavir plus self-peptide in K562-B*57:01, C1R-B*57:01, or autologous LCL systems. Abacavir nevertheless inhibited cognate EBV pHLA recognition, demonstrating that the drug can perturb B*57:01-restricted viral TCR-pHLA interactions. We interpret the absence of cross-recognition as a context-specific negative: it argues against those dominant EBV TCRs as drivers in those assay systems but does not globally exclude EBV or other herpesvirus epitopes from the model. Because the cellular site of abacavir hypersensitivity is not established, this limitation should not be converted into an unconstrained search across arbitrary cell types. Instead, future tests should use a finite, prespecified source-cell panel to identify abacavir-state B*57:01 self-peptides before testing paired microbial and altered-self pHLA recognition.

Consistent with this possibility, Almeida et al. showed that an HIV Gag TW10/HLA-B57-specific memory CD8 T-cell clone recognized autologous HLA-B57 only in the presence of abacavir; this is direct proof of principle for heterologous recognition, not evidence that HIV-specific T cells are the dominant clinical drivers of abacavir hypersensitivity [27]. Unlike some drug hypersensitivity settings in which public TCRs have been implicated [25], abacavir hypersensitivity has not been shown to be driven by a single public clonotype; available evidence is more consistent with broad, private, oligoclonal, or polyclonal recognition of an altered B*57:01 self-repertoire [22].

This helps explain why HLA class I is often necessary but rarely sufficient across phenotypes such as HLA-B*15:02–associated carbamazepine SJS/TEN (PPV ~3%), HLA-B*58:01 with allopurinol SJS/TEN and DRESS (PPV ~3%), HLA-B*13:01 with dapsone hypersensitivity (PPV ~7.8%), and HLA-B*57:01 with flucloxacillin drug-induced liver injury (PPV ~0.12%) [2].

Drug-reactive T cells can be engaged through multiple routes: (i) covalent haptenation, (ii) non-covalent repertoire alteration as in abacavir, and (iii) non-covalent drug-dependent recognition implicated in carbamazepine-associated SJS/TEN. These routes differ upstream but converge downstream on tissue recall and inhibitory threshold breach (Supplementary Figure S10.2).

### Why Drug Mimicry But Not Viral Exposure?

If persistent viral epitopes sustain durable effector T-cell memory in dynamic equilibrium with local tolerizing responses, why does exposure to the virus itself not routinely cause immunopathology, whereas drug-altered self sometimes does?

The answer lies in the difference between regulated equilibrium and acute perturbation (Figure 2). Under normal conditions, antiviral surveillance operates as a calibrated steady state: effector output is held below the tissue-damage threshold by PD-1, local regulatory T cells, and other inhibitory circuits, together with the limited density of viral antigen on any given cell surface [4, 15, 16]. This equilibrium has been tuned over the individual’s lifetime and, in evolutionary terms, over millions of years of host–virus co-adaptation.

Drug exposure can disrupt this equilibrium in several ways. First, a drug that alters a self-peptide can create a sudden, dense, tissue-localized display of mimicking ligands. Second, this display occurs on non-professional antigen-presenting cells (notably keratinocytes) that are poorly equipped to engage priming-stage tolerance mechanisms but are fully capable of activating pre-positioned TRM. Third, likely only one drug-altered peptide among many reproduces a specific geometry recognized by virus-trained TCRs. Fourth, only some individuals exposed to the drug arrive at TCR solutions that both recognize the viral epitope and cross-recognize the neo-epitope. Disease occurs when the composite mimicking burden—the product of ligand density, structural fidelity to the viral target, and niche match—exceeds the local inhibitory set-point. In contrast, viral exposure usually occurs within a long-established equilibrium between local effector and regulatory programs, whereas drug mimicry can present a denser and more abrupt ligand display in the same tissue niche.

### Herpesvirus Reactivation in DRESS

In drug reaction with eosinophilia and systemic symptoms (DRESS), herpesvirus reactivation is a recognized and clinically significant feature. DRESS is associated not only with HHV-6 reactivation—the most frequent and stereotyped signal, typically emerging 2–3 weeks after onset [28] — but often with sequential or concurrent reactivation of multiple human herpesviruses, including EBV, CMV, and HHV-7 [29–31]. In prospective cohorts, roughly 40–80% of DRESS patients show at least one viral reactivation, and more than half of those reactivate two or more viruses [30, 31]. Multiple reactivations are associated with more severe organ involvement and worse outcomes [30, 32]. Current data do not support a robust mapping between culprit drug and specific reactivating virus [30].

We propose the following model of this dynamic interplay (Figure 2, bottom row). Mimicry-driven immune engagement in one channel may be followed by broader compensatory tolerizing responses and diversion of antiviral attention, transiently loosening control over several latent herpesviruses in the same patient [4]. Which virus becomes clinically detectable may therefore depend more on host reservoir biology, disease severity, and timing than on a fixed drug–virus reactivation pairing. This is consistent with the observation that multiple herpesviruses reactivate in the same illness episode and argues against a strictly one-virus model of DRESS virology.

Herpesvirus reactivation in DRESS may also arise, at least in part, from immune activation itself, independent of direct epitope-specific mimicry. Tissue injury, eosinophilia, cytokine release, altered regulatory tone, and treatment timing can all reshape viral-load dynamics and lower thresholds for antigen-specific and bystander T-cell activation. This alternative is not incompatible with the model proposed here. A drug-triggered antigen-specific perturbation may initiate local immune disequilibrium, while broader inflammation and viral reactivation may amplify or sustain disease. Accordingly, herpesvirus reactivation in DRESS should be interpreted as a dynamic marker and potential amplifier of disrupted immune control, not as proof of a one-drug, one-virus mimicry event. Prior studies of DIHS/DRESS suggest dynamic regulatory T-cell changes during disease evolution, which may contribute to transient restraint, later immune dysregulation, or both [33].

Herpesvirus reactivation in DRESS cannot be reduced to a corticosteroid artifact: reactivation can precede corticosteroid administration [34, 35]. However, corticosteroid timing and dose shape the virologic pattern—early high-dose steroids may suppress HHV-6 reactivation, whereas CMV load can increase under corticosteroid exposure regardless of timing [35]. Steroids are therefore neither irrelevant nor a sufficient standalone explanation. EBV, CMV, and HHV-6 viral-load testing and ongoing monitoring are endorsed by recent international expert consensus on DRESS management and related guidance and may help identify patients at higher risk of flares or complications [36, 37]. Whether targeted antiviral therapy improves outcomes in DRESS merits prospective evaluation in mechanistically defined settings.

In contrast to DRESS, pure drug-induced SJS/TEN has not shown the same stereotyped pattern of sequential herpesvirus reactivation [38].

### Keratinocytes as Antigen-presenting Cells and Targets in SJS/TEN

**Figure 4.**
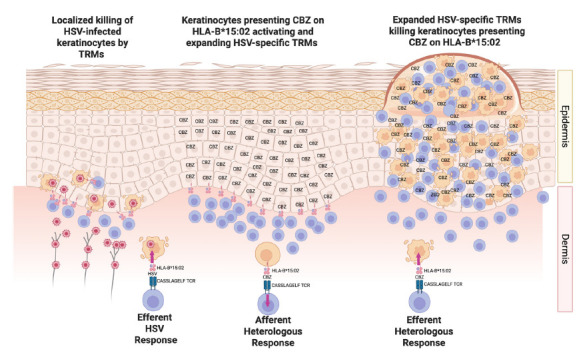
Proposed mechanism of HLA-B*15:02-associated carbamazepine hypersensitivity. Pre-existing tissue-resident memory T cells (TRM), originally specific for herpes simplex virus (HSV) [39], cross-recognize keratinocytes presenting carbamazepine (CBZ) as a neoantigen in the context of HLA-B*15:02. This leads to TRM activation and expansion and widespread keratinocyte apoptosis, culminating in severe epidermal damage characteristic of Stevens-Johnson syndrome (SJS). Created in BioRender. Phillips, E. (2025) https://BioRender.com/p27h658

In drug-induced SJS/TEN, keratinocytes paradoxically act as both antigen-presenting cells (APCs) and are direct targets of the cytotoxic attack they initiate (Figure 4) [39, 40]. Unlike professional APCs, keratinocytes are comparatively poor at inducing tolerogenic priming; instead, they present antigen in a tissue context where pre-positioned effector memory can be rapidly recruited if local inhibitory thresholds are breached. Drug-modified peptides that structurally resemble viral epitopes can trigger responses that outpace local tolerance mechanisms, particularly in individuals with preexisting HSV-specific TRM at the dermal-epidermal junction [39] (detailed keratinocyte evidence in Supplementary Material S7). Recent profiling of delayed-type drug hypersensitivity reactions has confirmed that systemic and skin-limited phenotypes associate with distinct resident versus recruited T-cell subsets [41], supporting the prediction that tissue-restricted injury reflects local TRM engagement rather than systemic immune activation.

### The Paradox of High Odds Ratios and Low PPV

Multiple independent but necessary factors explain the paradox of strikingly high odds ratios with high NPV but low PPV of HLA allelic associations [2] (Figure 3). HLA restriction provides an essential “double-hit” genetic framework. In the afferent phase, viral epitopes are presented by professional APCs via the risk HLA allele, initiating a coordinated immune response that primes CD4+ T cell help and yields a robust, immunodominant CD8+ tissue-resident memory response [39, 40]. On later drug exposure, the same allele presents a structurally similar drug-modified neoepitope, triggering cross-reactivity and driving local TRM expansion of the cross-reactive anti-viral T cells and pathogenic activation. In some settings, absence of a specific HLA allele yields very high NPV for a narrowly defined phenotype (for example, 100% for HLA-B*57:01 and immunologically confirmed abacavir hypersensitivity [1]). In others, such as HLA-B27 and ankylosing spondylitis, NPV is very high but not absolute, consistent with alternative alleles converging on similar sets of necessary conditions [42].

Variants in endoplasmic reticulum aminopeptidases (ERAP) further modulate peptide processing. Unfavorable ERAP allotypes increase the PPV of HLA-B27 for ankylosing spondylitis [42] and HLA-B*57:01 for abacavir hypersensitivity [43], presumably by altering peptide availability [22]. Population-scale virome data indicate that ERAP1/ERAP2 variation is associated with herpesvirus persistence [9], suggesting that these peptide-processing variants may modulate both viral load and drug-hypersensitivity risk. As additional necessary factors are identified (HLA, viral epitope, TCR, drug), PPV increases, progressively defining the collective requirements for disease.

Heterologous immunity, in which pre-existing virus-specific memory T cells cross-react with novel antigens, is another crucial factor [5, 44]. This principle underlies our hypothesis that HSV- or VZV-specific TRM restricted by HLA-B*15:02 and bearing the public TCR-beta CDR3 ASSLAGELF clonotype mediate carbamazepine-induced SJS/TEN [5, 21, 25] (Figure 4). However, it should be emphasized that any virus-trained TCR, private or public, not just public TCRs, may result in hypersensitivity if an altered self-peptide provides a structural mimic of sufficient density.

## EBV AND MULTIPLE SCLEROSIS

EBV provides a well-documented example of how persistent viral infection may contribute to organ-specific autoimmunity. MS patients are almost universally EBV-seropositive [45], and high IgG titers to the EBV latent protein EBNA1 correlate with increased MS risk [46]. Epidemiologic data consistently show that delayed or severe primary EBV infection (infectious mononucleosis) increases MS risk [45, 46]. Early childhood infection may establish a narrower, more adaptive dynamic equilibrium with EBV than the intense inflammatory context of primary infection in adolescence or later (Supplementary Material S8 and Supplementary Figure S10.3).

Recent mechanistic work has strengthened the case for a direct EBV–MS link. Wang et al [47] demonstrated that EBV infection alters the peptide repertoire presented by HLA-DR15 (the strongest MS genetic risk factor), enabling presentation of myelin-derived peptides that would otherwise be poorly represented in the groove. This provides evidence that EBV changes what the risk HLA can display, rather than acting solely through bystander activation. Complementing this, Thomas et al [48] identified cross-reactive T cells recognizing both EBNA1 and the CNS ion channel anoctamin-2 (ANO2) in MS patients, providing mechanistic support for molecular mimicry between an EBV antigen and a CNS autoantigen. These T cells were expanded in MS patients compared with healthy EBV-seropositive controls, suggesting disease-specific amplification of the cross-reactive compartment. However, single-cell analysis of clonally enriched CD8+ T cells in MS lesions identified EBV-specific clonotypes in the CNS compartment but did not demonstrate self-cross-reactivity in the clones tested [49]. This negative result should be interpreted cautiously: only a limited number of clonotypes could be assayed, and the full repertoire of potentially relevant modified-self ligands could not be exhaustively tested. Moreover, once a cross-reactive response initiates tissue injury, secondary epitope spreading may broaden the lesion response and obscure the initiating event. These considerations suggest that compartmentalized EBV-directed CD8 responses and CD4-mediated molecular mimicry may operate through parallel rather than identical pathways, without excluding a role for CD8 cross-reactivity that current sampling has not captured.

Immune checkpoint inhibitor (ICI) therapy further reveals this balance: ICIs can unmask pre-existing virus-directed T-cell responses, sometimes triggering severe tissue damage as seen in ICI-induced EBV-driven encephalitis [50]. Such toxicities are best interpreted as unmasking of entrenched memory rather than *de novo* autoimmunity [7, 51].

## TRANSPLANTATION

Allo-recognition offers a controlled probe for heterologous immunity. In the HLA-B*08:01-FL-RGRAYGL/HLA-B*44:02 system, EBV-specific CTLs from healthy EBV-exposed donors cross-recognized HLA-B*44:02 in vitro, and this cross-reactivity dominated the anti-HLA-B*44:02 response in the donors studied [52]. More broadly, systematic screening of virus-specific memory T cells against HLA panels found allo-HLA cross-reactivity in 80% of EBV-, CMV-, VZV-, and influenza-specific T-cell lines and 45% of individual virus-specific T-cell clones [53]. Public allo-HLA cross-reactivities shared across unrelated individuals have also been demonstrated [54]. These findings show that virus-specific memory can contain substantial pre-existing allo-HLA reactivity, with potential relevance to transplantation [55].

## EXPERIMENTAL VALIDATION

The central predictions are testable but require approaches matched to the model’s complexity.

### What Can Be Tested Now in Humans

**Figure 5.**
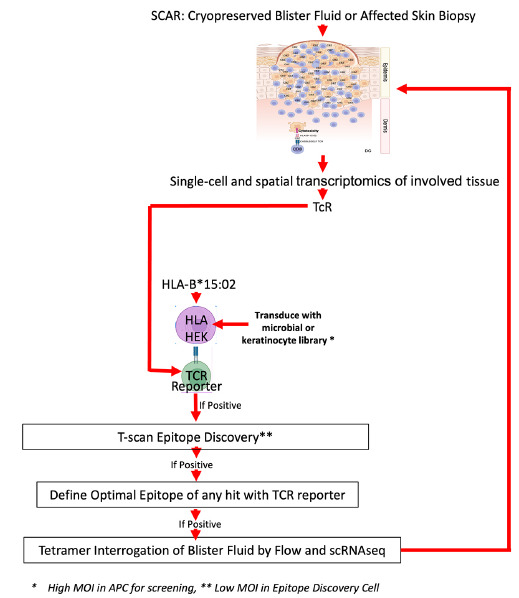
**Lesion-anchored roadmap for TCR-based epitope discovery and validation in drug hypersensitivity.** Starting from cryopreserved blister fluid or affected skin biopsy obtained at the time and site of pathology, single-cell transcriptomic profiling identifies expanded lesional clonotypes and nominates candidate disease-relevant TCRs. Selected TCRs are then reconstructed in a reporter system together with the relevant HLA restriction element, illustrated here with HLA-B*15:02, and screened against microbial or keratinocyte-focused libraries to identify activating ligands. Positive hits are refined to define the minimal activating epitope, after which peptide–HLA tetramers are generated and used to re-probe the original lesional material by flow cytometry and single-cell analysis. This closed-loop workflow moves from lesion-derived clonotype to candidate viral epitope or modified-self mimic and back to the original pathology specimen for confirmation, thereby reducing candidate-antigen bias and directly testing cross-recognition in the relevant tissue niche. *High multiplicity of infection in APC-based screening step. **Lower multiplicity in the epitope-discovery cell used for secondary mapping.

The decisive experiment is to determine whether lesion-enriched T-cell clonotypes cross-recognize both drug-modified self and viral peptides in the same individuals. A lesion-anchored roadmap for this strategy is shown in Figure 5. The key tactical choice is to treat the lesion as the ground truth for the relevant tissue niche: single-cell and spatial profiling identify the dominant clonotypes at the time and site of pathology; selected TCRs are then reconstructed in reporter systems for staged epitope-discovery screens against viral and keratinocyte-focused libraries; and candidate ligands are taken back to the original specimen for tetramer-based re-probing and orthogonal confirmation. Scalable TCR synthesis and epitope-discovery screening platforms [56] now make this approach practical at higher throughput. Any putative cross-reactive circuit would still require demonstration that the candidate ligands are naturally processed and presented on the relevant target cell, together with independent biophysical confirmation of dual recognition by the same TCR, for example, using surface plasmon resonance or single-molecule dual optical tweezer assays.

Although illustrated here with drug hypersensitivity examples, the same lesion-first strategy is generalizable to T cell–mediated hypersensitivity and autoimmunity more broadly; the key requirement is to obtain disease-relevant T cells at the time and site of pathology, before epitope spreading obscures the initiating response.

In practice, combined evidence of oligoclonal TCR expansion and a transcriptomic signature of cytotoxicity within a lesion-associated subcluster can help prioritize at least four or five TCRs for investigation. One route is unbiased epitope discovery, as shown in Figure 5.

A complementary route is candidate-based: once the keystone epitope is discovered, one can sometimes rapidly nominate a candidate self-peptide that binds, or is predicted to bind, the risk HLA allele and has sufficient homology to the keystone epitope to be a plausible cross-reactive target. Both approaches may converge on the same initiating self-peptide. This is most productive before epitope spreading broadens the lesion repertoire and makes discovery more difficult; at later stages, the relevant self-peptide may be recognized in its native rather than drug-modified form.

Cases of drug-induced hypersensitivity can be compared with drug-tolerant controls to test whether viral serostatus, HLA genotype, ERAP variation, and germline TCR are jointly necessary risk factors, as predicted by the model.

Abacavir hypersensitivity illustrates a complementary, candidate-pair-first strategy. Because HLA-B*57:01 screening has greatly reduced incident abacavir hypersensitivity, this approach is most realistic using archived samples, PBMCs, patch-test material, or other available material from individuals with prior abacavir hypersensitivity. Because the cellular site of abacavir hypersensitivity is not established, candidate abacavir-state B*57:01 self-peptides should be nominated from a finite, prespecified discovery panel rather than inferred retrospectively after negative results. A defensible first panel would include B-lineage cells or LCLs, monocytes or monocyte-derived macrophages, monocyte-derived dendritic cells, and endothelial cells, with additional contexts added only by explicit prospective rationale. Candidate self-peptides should be prioritized by drug-dependent B*57:01 presentation, small hydrophobic C-terminal anchor compatibility, natural-processing plausibility, expression in the prespecified source-cell panel, and TCR-facing similarity to B*57:01-restricted microbial epitopes. Paired tetramers can then be generated: one fluorochrome for the candidate microbial pHLA and another for the abacavir-dependent self-peptide-HLA-B*57:01 complex. Dual-positive CD8 T cells can be single-cell sorted for paired TCR alpha/beta sequencing, followed by TCR reconstruction and testing of both ligands in controlled single-HLA-B*57:01 systems. This directly tests whether rare memory T cells recognize both a microbial pHLA and a specific abacavir-state self-pHLA surface.

The AHS-specific keystone-epitope prediction would be substantially weakened if a prespecified source-cell panel fails to identify abacavir-state B*57:01 self-peptides capable of presentation and T-cell recognition; if a prespecified panel of dominant B*57:01-restricted herpesvirus, HIV where relevant, and other microbial epitopes fails to identify dual-positive CD8 T cells in available AHS patient material; and if reconstructed candidate TCRs fail to recognize both the microbial pHLA and the abacavir-state self-pHLA under controlled single-HLA-B*57:01 conditions. Negative results should therefore be reported with their assay context, rather than treated either as global exclusion or ignored as uninformative.

### What Can Be Tested in Animal Models

Animal models are best used as hypothesis-testing tools after the likely mechanistic basis has been determined from human studies. For example, if human data identify a specific drug, HLA allele, and TCR clonotype that cross-recognizes both a viral epitope and a drug-modified self-peptide, those defined elements can be engineered into a transgenic or humanized mouse system to test whether prior infection with the relevant virus is necessary to recapitulate disease. Existing models include HLA-transgenic mice (eg, the HLA-B*57:01 abacavir model [24]), murine CMV systems that can test whether prior viral imprinting shapes responses to subsequent neoantigen challenge, and humanized mouse models that can reconstitute human TCR–pHLA interactions in a controlled background.

### What Animal Models Cannot Recapitulate

No animal model can fully recapitulate the relevant polymorphic human HLA, KIR, NK receptors, and T-cell architecture together with the diversity of potential priming human-adapted organisms. Therefore, no single preclinical animal model can identify all clinically relevant drug hypersensitivity syndromes before first human use. These limitations underscore why human studies remain the primary discovery pathway and why animal models can only be designed once the necessary disease-mediating elements are known from human data.

## PRACTICAL IMPLICATIONS

### Risk Prediction

If only certain peptide–HLA geometries engage entrenched viral memory, risk prediction should focus on niches where modified self plausibly mimics these responses. Potential modifiers such as ERAP variation, KIR/HLA combinations, germline TCRs, and HLA-C expression levels can be weighed in context and investigation prioritized toward the oligoclonally expanded TCRs that dominate at the site and time of pathology.

### Monitoring

Tracking herpesvirus reactivation may serve as a practical proxy for periods when immune hierarchies are under stress—for example, during severe hypersensitivity or early post-transplant immunosuppression. This remains a testable proposition requiring prospective validation.

### Mitigation

Viral reactivation in drug hypersensitivity may reflect tolerizing responses to mimicry-driven immune engagement, not only nonspecific immune suppression. Where virological footprints accompany immunopathology, combining targeted antiviral therapy with immune modulation to prevent secondary DRESS flares may merit prospective evaluation in mechanistically defined settings.

## CONCLUSION

Several observations are well established: HLA class I risk alleles are necessary but not sufficient for T cell–mediated drug hypersensitivity; persistent herpesviruses seed tissue-resident memory in specific anatomical niches; ERAP variation modifies both viral persistence and HLA-disease associations; and T cells recognizing both EBNA1 and ANO2 have been identified in MS patients [48]. We propose that these observations may be connected—that immune focusing by persistent viruses, while protective, may, in rare circumstances, create conditions in which structural neo-antigens that approximate entrenched viral targets recruit tissue-positioned memory against self. If correct, this would help account for the high NPV but low PPV of HLA risk alleles and the tissue-restricted patterning of injury. A decisive human test would demonstrate that T-cell clonotypes enriched at a drug hypersensitivity lesion site cross-react with both the drug neoepitope and a viral peptide in the same individual, with orthogonal confirmation of natural processing and dual recognition. An additional prediction is that once tissue injury begins, the lesion response may broaden to include unmodified self-peptides through epitope spreading, potentially obscuring the initiating cross-reactive event. Scalable TCR-to-antigen mapping platforms [56] now make these experiments feasible. The same lesion-anchored strategy—identifying disease-relevant TCRs at the site of pathology, discovering the keystone epitope, and nominating or discovering the initiating self-peptide before epitope spreading obscures the signal—may be generalizable to T cell–mediated hypersensitivity and autoimmunity more broadly. The same discovery logic may also run in the other direction. Applied to rapidly evolving RNA viruses and tumors, TCR-anchored epitope discovery could distinguish constrained targets capable of engaging keystone-imprinted memory productively from the mutable decoy epitopes that capture immunodominance without conferring control, a distinction with direct consequences for immunogen selection and one we develop in a companion review in this issue [57].

## SUPPLEMENTARY DATA

Supplementary materials are available at the *Pathogens and Immunity* website. Supplementary data may be provided by the authors to benefit the reader. Supplementary data are not copyedited and are the sole responsibility of the authors. Questions or comments related to supplementary materials should be addressed to the corresponding author.
