# Supplementary material for: Keystone Epitope Theory: Implications for Hypersensitivity, Autoimmunity, and Transplantation
Source: Pathog Immun. 2026 Aug 7;11(2):16–38. doi: 10.20411/pai.v11i2.1016 (PMC13471026; doi:10.20411/pai.v11i2.1016)
Supplement: Supplementary file 1 [file pai-11-016-s01.pdf]

PATHOGENS AND IMMUNITY
IN ASSOCIATION WITH CASE WESTERN RESERVE UNIVERSITY AND THE RICHARD J. FASENMYER FOUNDATION

Supplementary Materials

Contents

S1. Glossary 1

S2. Supplementary Table S1 2

S3. Evidence for Herpesvirus-Associated Heterologous Immunity 2

S4. NK Cell–HLA Coordination 2

S5. Structural Basis for TCR Cross-Reactivity 3

S6. IgE-Mediated Allergy vs T Cell–Mediated Hypersensitivity 3

S7. Detailed Keratinocyte Evidence in SJS/TEN 3

S8. Exposure Timing: Selected Observations and Caveats 3

S9. Experimental Validation: Workflow Details and Animal-Model Limits 4

S9.1 Lesion-to-mechanism workflow *4*

S9.2 What reduced animal systems can test *5*

S9.3 What animal models cannot recapitulate *5*

S10. Supplementary Figures 6

References 8

S1. GLOSSARY

pHLA (peptide–HLA). The peptide–HLA complex on the cell surface, read by CD8 TCRs and, in some contexts, by NK receptors via KIR and NKG2.

TRM (tissue-resident memory T cell). A memory T-cell population that persists in tissues and responds rapidly in situ without recruitment from blood.

Modified self-ligand. A self-derived pHLA complex perceived as non-self because the peptide repertoire is altered by a drug or post-translational modification.

Heterologous immunity. Prior pathogen-specific memory shapes responses to an unrelated antigen, often via TCR cross-reactivity.

Public / private TCR. Public TCR solutions are shared across multiple individuals; private TCRs are individual-specific.

NPV / PPV. Negative and positive predictive value. Low PPV under strong HLA associations implies additional necessary conditions beyond HLA.

ERAP. Endoplasmic reticulum aminopeptidases shape which peptides are available for HLA class I presentation.

 Mallal S, Asiaee A, Phillips E. Keystone Epitope Theory: Implications for Hypersensitivity, Autoimmunity, and Transplantation. *Pathogens and Immunity*. 2026;11(2):16-38. doi: 10.20411/pai.v11i2.1016

Keystone organism / epitope. As used in this review: a persistent, niche-structured pathogen (eg, CMV, EBV, HSV) whose conserved epitopes seed durable, tissue-positioned T-cell memory through repeated antigenic stimulation [1].

S2. SUPPLEMENTARY TABLE S1

Supplementary Table S1. Operational features used in this review to characterize persistent organisms and their epitopes. This is a heuristic summary, not a formal taxonomy.

Feature	Organism level	Epitope level
Chronic persistence and reactivation	Establish lifelong latency with intermittent reactivation	Sustain durable T-cell memory through periodic antigenic restimulation
Tissue/niche restriction	Seed TRM in defined niches (eg, HSV at dermal–epidermal junction, EBV in lymphoid tissue, CMV in vascular endothelium)	Presented in anatomical contexts that shape compartment-specific immune coordination
Host-regulated viral burden	Viral DNA burden is a measurable, host-regulated trait shaped by antigen processing and MHC genotype [2, 3]	Constrained by host peptide-processing machinery (ERAP1/2, LNPEP, tapasin)
Functionally constrained targets	Organisms for which immune escape mutations carry a replicative fitness cost	Epitopes where amino acid substitution impairs viral fitness, favoring durable immune targeting

S3. EVIDENCE FOR HERPESVIRUS-ASSOCIATED HETEROLOGOUS IMMUNITY

Persistent herpesvirus infections have been associated with enhanced immune responses to unrelated pathogens. CMV-seropositive individuals show augmented CD8+ T-cell effector function in response to heterologous viral antigens such as influenza [4]. In HSV models, the cited evidence is indirect and concerns tissue positioning and regulation rather than demonstrated heterologous protection. LAG-3 blockade combined with therapeutic vaccination restored tissue-resident antiviral CD8+ T-cell function and improved control of recurrent ocular HSV [5]. Ultraviolet-inactivated HSV-1 shifted an atopic dermatitis-like response toward a Th1 pattern [6]. Regulatory T cells modulated vaccine-generated primary and memory HSV-specific CD8+ T-cell responses [7]. A laser-adjuvanted HSV peptide vaccine mobilized skin dendritic cells and induced protective local effector-memory and tissue-resident-memory CD8+ T-cell responses [8]. These findings are contingent on timing and host genetic context. They are consistent with, but do not prove, the hypothesis that persistent herpesviruses contribute to broader immune calibration.

S4. NK CELL–HLA COORDINATION

A single pHLA complex can be read by both CD8 T cells (via TCR) and NK cells (via KIR and CD94–NKG2 receptors). Peptide-specific engagement of NK-cell inhibitory and activating receptors tunes NK-cell responses depending on the precise HLA-peptide combination [9]. Changes in the presented peptide repertoire—from viral adaptation, drug exposure, or altered processing— can differentially affect CTL

and NK thresholds. Loss of CTL visibility through HLA downregulation may simultaneously disinhibit NK cells, and vice versa. This dual-reading architecture helps explain why some drug-altered peptidomes trigger CTL-mediated injury without disinhibiting NK cells, and why KIR/HLA genotype modifies disease risk and severity.

S5. STRUCTURAL BASIS FOR TCR CROSS-REACTIVITY

TCR cross-reactivity is relatively common, reflecting the high frequency of shared TCR clonotypes across individuals and the structural constraints on TCR diversity [10]. Many TCRs recognize multiple structurally similar but distinct epitopes, including pathogen-derived antigens and self-peptides modified by drugs or post-translational modifications. Immune tolerance mechanisms regulate most cross-reactive interactions, but virus-specific TCRs that form part of a tissue-positioned memory response may be activated by mimicking ligands that breach local regulatory thresholds.

Shared TCR clonotypes are common across individuals[10], and HLA class I binding is related to evolutionary conservation in human and viral proteins [11]. These observations provide a plausible substrate for cross-reactivity but do not themselves demonstrate herpesvirus-specific recognition of modified self. Some longer HLA class I-presented viral peptides bulge from the binding groove. In the EBV example studied by Tynan et al, a relatively rigid TCR face flattened the bulged peptide, with conformational adjustment occurring predominantly in the peptide rather than the TCR [12]. This illustrates one route by which distinct peptides could converge on a similar TCR-facing surface, but it does not itself establish cross-reactivity with modified self.

S6. IGE-MEDIATED ALLERGY VS T CELL–MEDIATED HYPERSENSITIVITY

IgE-mediated allergies and T cell–mediated drug hypersensitivity differ in their mechanisms and clinical trajectories. IgE-mediated food and drug allergies typically arise from disrupted mucosal tolerance and are often short-lived, route-dependent, and amenable to desensitization [13-17]. T cell–mediated hypersensitivity, by contrast, may arise when drug-modified peptides engage preexisting, tissue-positioned memory T cells originally trained on persistent viral epitopes. Because these effectors are maintained by ongoing viral surveillance rather than classical peripheral tolerance, the resulting responses can be durable [18]. This distinction may be relevant to clinical counseling: IgE-mediated reactions involve route and barrier considerations, whereas T cell–mediated reactions involve HLA genotype, tissue niche, and prior viral exposure.

S7. DETAILED KERATINOCYTE EVIDENCE IN SJS/TEN

For the non-covalent drug-dependent recognition route, sustained exposure to an offending drug can create a reversible antigenic surface on peptide-loaded risk HLA expressed by keratinocytes, activating pre-existing CD8+ TRM in the skin. This illustrates the non-covalent, drug-dependent recognition route to Signal 1 and contrasts with covalent haptenation and with abacavir-like repertoire alteration (Figure S10.2). Activation may proceed with reduced reliance on de novo priming when sufficient drug-dependent Signal 1, together with tissue context, breaches local inhibitory thresholds.

Once activated, these CD8+ TRM recognize and target keratinocytes presenting drug neoepitopes via the risk HLA allele. This leads to widespread keratinocyte apoptosis and subsequent epidermal necrolysis, as well as damage to other mucosal surfaces, including corneal epithelium, oral mucosa, and genital tract [19]. This is consistent with why the disease is severe, localized to drug-exposed epithelial surfaces, progresses rapidly even after drug discontinuation, and can recur upon re-exposure years later.

S8. EXPOSURE TIMING: SELECTED OBSERVATIONS AND CAVEATS

Selected cohort studies report associations between early herpesvirus acquisition and reduced rates of allergic and autoimmune disease. In the Rhea cohort (Greece), children seropositive for multiple

herpesviruses by age 6 were less likely to develop eczema [20]. In the Generation R cohort (Netherlands), children with serological evidence of prior CMV, EBV, or HSV-1 infection were less likely to test positive for transglutaminase type 2 antibodies, a marker of celiac autoimmunity [21]. In comparative studies of farming communities, children raised in higher-exposure settings show lower rates of asthma and allergic sensitization than genetically similar children in lower-exposure settings [22, 23]. Epidemiologic associations between delayed EBV acquisition and increased MS risk are well documented [24, 25], though the causal pathway is not resolved.

Caveats. These observations are associative, not proof of causation. Multiple confounders (diet, household size, antibiotic use, microbial diversity) covary with the timing of herpesvirus acquisition. The overwhelming population-level benefits of improved sanitation, vaccination, antibiotics, safer childbirth, shelter, and nutrition are not in question. Any exposure-timing hypothesis concerns rare immune tradeoffs among individuals who survive and thrive under modern conditions. Autoimmune disease and drug hypersensitivity remain minority outcomes requiring additional genetic and immunological gates beyond exposure timing alone.

S9. EXPERIMENTAL VALIDATION: WORKFLOW DETAILS AND ANIMAL-MODEL LIMITS

S9.1 Lesion-to-mechanism workflow

Figure 5 in the main text summarizes this lesion-to-mechanism workflow schematically; the steps below expand that logic in prose.

The central prediction is that T-cell clonotypes enriched at the site of drug hypersensitivity cross-recognize both the drug-modified self-peptide and a viral peptide presented by the same HLA allele. Testing this requires a staged workflow:

1. **Lesion-enriched clonotype identification.** Single-cell TCR sequencing of T cells from the active lesion (eg, blister fluid in SJS/TEN, skin biopsy in DRESS) identifies oligoclonally expanded TCR$\alpha\beta$ pairs.
2. **Cognate pHLA ligand discovery.** Candidate drug-modified peptides are identified by HLA peptidomics of drug-exposed cells bearing the risk allele. Peptide–HLA tetramers confirm binding by the lesion-enriched clonotypes.
3. **Cross-recognition testing.** The same clonotypes are tested for recognition of candidate viral peptides (eg, HSV, VZV, or EBV epitopes restricted by the same HLA allele) using tetramer staining, cytotoxicity assays, or activation readouts.
4. **Orthogonal confirmation.** Candidate cross-reactive peptides must be confirmed to be naturally processed and presented on the relevant cell types (keratinocytes for SJS/TEN; hepatocytes for DILI) under physiologic conditions. Independent biophysical confirmation of dual recognition by the same TCR would further strengthen the finding.

Scalable TCR synthesis and epitope-discovery screening platforms [26] now permit systematic mapping of TCR-to-antigen specificities, making high-throughput cross-reactivity testing feasible. Prospective cohorts that combine viral serostatus, HLA genotype, ERAP genotype, and drug-challenge outcomes can test whether the predicted risk factors are jointly necessary.

S9.2 What reduced animal systems can test

- **HLA-transgenic mice** (eg, the HLA-B*57:01 abacavir model [27]) can test whether drug-modified peptidomes generate T cells that cross-react with viral peptides restricted by the same HLA.
- **Murine CMV models** can test whether prior viral imprinting biases T-cell responses to a subsequent neoantigen challenge and whether the temporal sequence matters.
- **Humanized mouse models** can reconstitute human TCR–pHLA interactions in a controlled background, permitting direct testing of human clonotype cross-reactivity.

S9.3 What animal models cannot recapitulate

- **Immune receptor polymorphism.** Inbred mice lack the HLA, KIR, and NKG2 diversity that defines human disease susceptibility.
- **Natural viral acquisition.** Under standard laboratory conditions, mice do not naturally transmit murine CMV; infection requires artificial inoculation.
- **Lifelong co-adaptation.** No animal model recapitulates decades of intermittent herpesvirus reactivation in a polymorphically diverse host.
- **Multi-gate causality.** Testing whether a disease requires the simultaneous satisfaction of multiple independent variable conditions requires the genetic and viral diversity of human populations.

These limitations underscore why human lesion-based and cohort-based approaches remain the primary validation path.

PATHOGENS AND IMMUNITY
IN ASSOCIATION WITH CASE WESTERN RESERVE UNIVERSITY AND THE RICHARD J. FASENMYER FOUNDATION

S10. SUPPLEMENTARY FIGURES

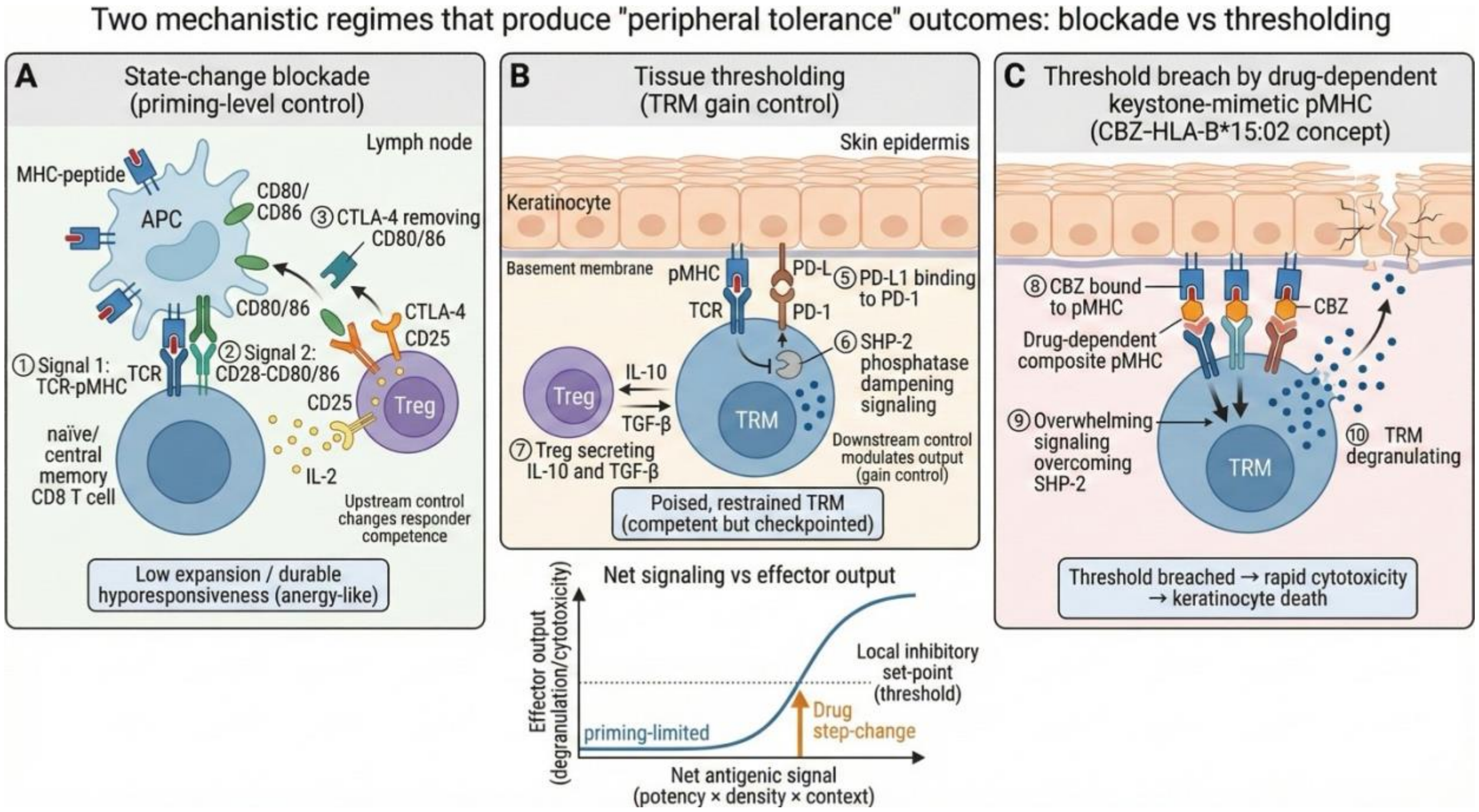

Figure S10.1. Two regulatory regimes that can produce clinical tolerance: priming-stage blockade versus tissue-level thresholding.

(A) Priming-stage blockade operates at costimulatory checkpoints, where antigen recognition occurs but clonal expansion and durable competence are constrained. **(B)** Tissue-level thresholding operates within barrier niches containing TRM; inhibitory receptors impose local gain control so that competent TRM remain poised yet restrained until net signaling exceeds the local inhibitory set-point. **(C)** In a drug-dependent mimicry scenario, a step-change in composite pHLA signaling may breach the threshold, converting poised restraint into rapid cytotoxicity.

Mallal S, Asiaee A, Phillips E. Keystone Epitope Theory: Implications for Hypersensitivity, Autoimmunity, and Transplantation. *Pathogens and Immunity*. 2026;11(2):16-38. doi: 10.20411/pai.v11i2.1016

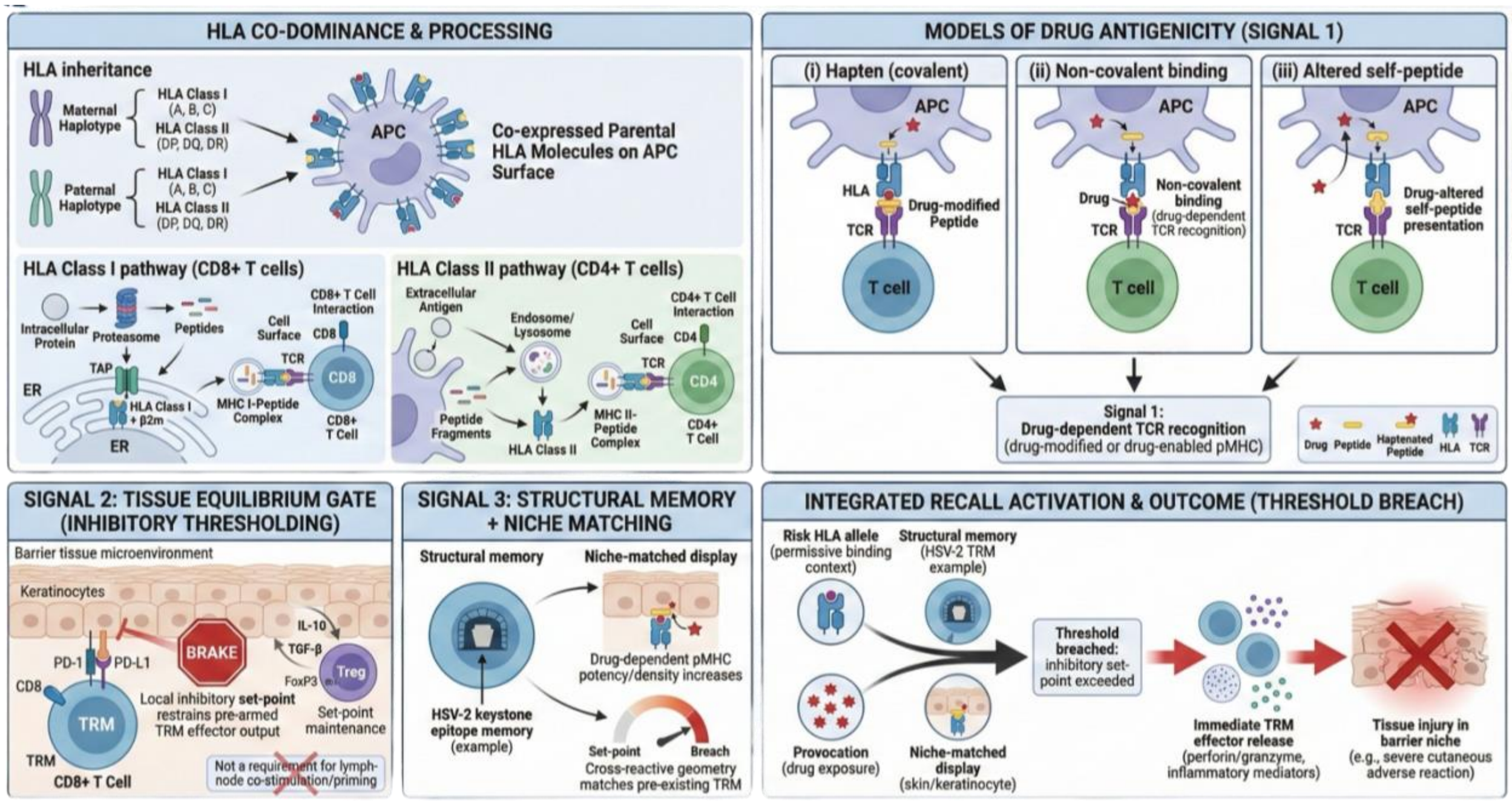

Figure S10.2. Routes to drug antigenicity converge on tissue-level threshold breach.

Overview of how distinct chemical routes to drug antigenicity converge on shared tissue-level pathophysiology. Top left: HLA co-dominant inheritance and antigen processing shape the peptide–HLA landscape. Top right (Signal 1): three models of drug antigenicity: (1) hapten/prohapten, involving a covalent adduct on a self-peptide; (2) non-covalent drug-dependent recognition, as in carbamazepine-associated SJS/TEN, in which a reversible drug interaction creates a transient antigenic surface; and (3) altered peptide repertoire, as in abacavir exposure, in which non-covalent drug binding shifts the presented self-peptides. Bottom left (Signal 2): tissue equilibrium gate with local inhibitory pathways setting an activation threshold. Bottom middle: tissue-resident memory niche matching. Bottom right: integrated model in which sufficient net signaling breaches inhibitory set-points, enabling rapid tissue recall and tissue-specific injury.

Abbreviations: HLA, human leukocyte antigen; TCR, T-cell receptor; APC, antigen-presenting cell; TRM, tissue-resident memory T cell; SJS/TEN, Stevens–Johnson syndrome/toxic epidermal necrolysis.

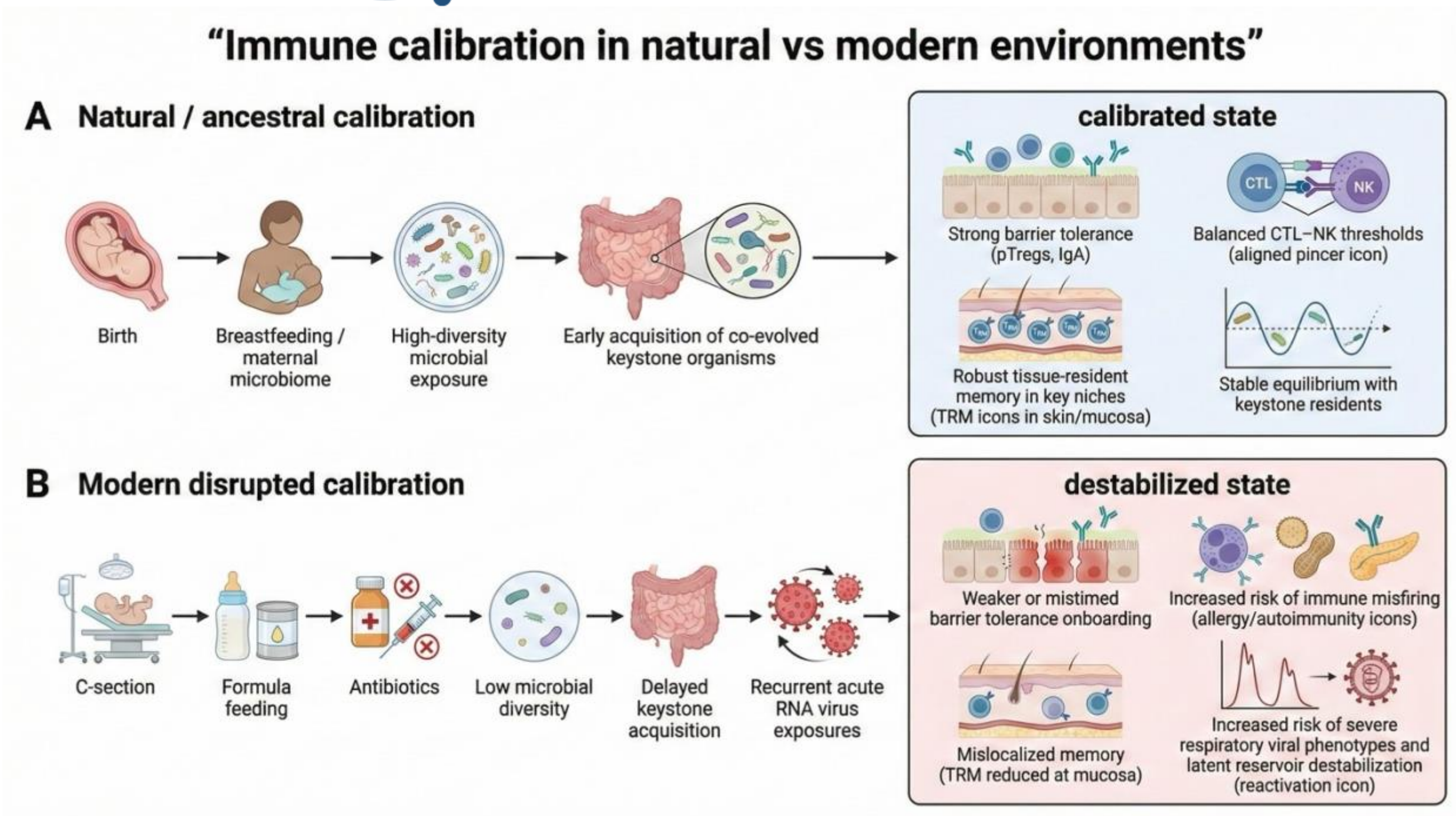

Figure S10.3. Exposure timing: immune calibration in different epidemiologic settings. In settings with earlier acquisition of persistent infections, immune development may be shaped through structured imprinting. In modern settings, delayed acquisition of persistent viruses and reduced early-life microbial diversity may alter immune calibration. **Important:** this figure illustrates possible rare immune tradeoffs among individuals who survive and thrive under modern conditions. The overwhelming population-level benefits of modern sanitation, vaccination, antibiotics, safer childbirth, shelter, and nutrition are not in question and are not criticized by this model.

REFERENCES

1. Asiaee A, Mallal N, Phillips E, Mallal S. Co-Evolved Partners of Immunity: A Trait-Based Map of Human Keystone Organisms. *bioRxiv*. 2025. doi: 10.1101/2025.08.19.671142. PubMed PMID: 41502936; PMCID: PMC12773006.
2. Nyeo SS, Cumming EM, Burren OS, Pagadala MS, Gutierrez JC, Ali TA, Kida LC, Chen Y, Chu H, Hu F, Zou XZ, Hollis B, Fabre MA, MacArthur S, Wang Q, Ludwig LS, Dey KK, Petrovski S, Dhindsa RS, Lareau CA. Population-Scale Sequencing Resolves Determinants of Persistent EBV DNA. *Nature*. 2026;650(8102):664–72. doi: 10.1038/s41586-025-10020-2.
3. Kamitaki N, Tang D, McCarroll SA, Loh P-R. The DNA Virome Varies with Human Genes and Environments. *bioRxiv*. 2026:2025.09.08.674901. doi: 10.1101/2025.09.08.674901.
4. Furman D, Jojic V, Sharma S, Shen-Orr SS, Angel CJ, Onengut-Gumuscu S, Kidd BA, Maecker HT, Concannon P, Dekker CL, Thomas PG, Davis MM. Cytomegalovirus Infection Enhances the Immune Response to Influenza. *Sci Transl Med*. 2015;7(281):281ra43. doi: 10.1126/scitranslmed.aaa2293. PubMed PMID: 25834109; PMCID: PMC4505610.
5. Roy S, Coulon PG, Srivastava R, Vahed H, Kim GJ, Walia SS, Yamada T, Fouladi MA, Ly VT, BenMohamed L. Blockade of LAG-3 Immune Checkpoint Combined With Therapeutic Vaccination Restore the Function of Tissue-Resident Anti-Viral CD8+ T Cells and Protect Against Recurrent

Ocular Herpes Simplex Infection and Disease. *Front Immunol*. 2018;9:2922. doi: 10.3389/fimmu.2018.02922. PubMed PMID: 30619285; PMCID: PMC6304367.

6. Horiuchi Y, Bae S, Katayama I, Mori Y, Yamanishi K. Effects of Ultraviolet-Inactivated Herpes Simplex Virus Type I on Atopic Dermatitis-Like Lesions in NC/Nga Mice: Role of the Suppressor of Cytokine Signaling in the Skin. *Allergology International*. 2004;53(4):331–40. doi: 10.1111/j.1440-1592.2004.00360.x.
7. Toka FN, Suvas S, Rouse BT. CD4+CD25+ T Cells Regulate Vaccine-Generated Primary and Memory CD8+ T-Cell Responses against Herpes Simplex Virus Type 1. *Journal of Virology*. 2004;78(23):13082–9. doi: 10.1128/jvi.78.23.13082-13089.2004.
8. Lopes PP, Todorov G, Pham TT, Nesburn AB, Bahraoui E, BenMohamed L. Laser Adjuvant-Assisted Peptide Vaccine Promotes Skin Mobilization of Dendritic Cells and Enhances Protective CD8+ TEM and TRM Cell Responses against Herpesvirus Infection and Disease. J Virol. 2018;92(8):e02156-17. doi: 10.1128/JVI.02156-17. PubMed PMID: 29437976; PMCID: PMC5874417.
9. Sim MJW, Long EO. The Peptide Selectivity Model: Interpreting NK Cell KIR-HLA-I Binding Interactions and Their Associations to Human Diseases. *Trends Immunol*. 2024;45(12):959–70. doi: 10.1016/j.it.2024.10.006. PubMed PMID: 39578117.
10. Soto C, Bombardi RG, Kozhevnikov M, Sinkovits RS, Chen EC, Branchizio A, Kose N, Day SB, Pilkinton M, Gujral M, Mallal S, Crowe JE, Jr. High Frequency of Shared Clonotypes in Human T Cell Receptor Repertoires. *Cell Rep*. 2020;32(2):107882. doi: 10.1016/j.celrep.2020.107882. PubMed PMID: 32668251.
11. Hertz T, Nolan D, James I, John M, Gaudieri S, Phillips E, Huang JC, Riadi G, Mallal S, Jojic N. Mapping the Landscape of Host-Pathogen Coevolution: HLA Class I Binding and Its Relationship with Evolutionary Conservation in Human and Viral Proteins. *J Virol*. 2011;85(3):1310–21. doi: 10.1128/JVI.01966-10. PubMed PMID: 21084470; PMCID: PMC3020499.
12. Tynan FE, Reid HH, Kjer-Nielsen L, Miles JJ, Wilce MC, Kostenko L, Borg NA, Williamson NA, Beddoe T, Purcell AW, Burrows SR, McCluskey J, Rossjohn J. A T Cell Receptor Flattens a Bulged Antigenic Peptide Presented by a Major Histocompatibility Complex Class I Molecule. *Nat Immunol*. 2007;8(3):268–76. doi: 10.1038/ni1432. PubMed PMID: 17259989.
13. Gomez de Agüero M, Ganal-Vonarburg SC, Fuhrer T, Rupp S, Uchimura Y, Li H, Steinert A, Heikenwalder M, Hapfelmeier S, Sauer U, McCoy KD, Macpherson AJ. The Maternal Microbiota Drives Early Postnatal Innate Immune Development. *Science*. 2016;351(6279):1296–302. doi: 10.1126/science.aad2571.
14. Renz H, Blümer N, Virna S, Sel S, Garn H. The Immunological Basis of the Hygiene Hypothesis. *Chem Immunol Allergy*. 2006;91:30–48. doi: 10.1159/000090228. PubMed PMID: 16354947.
15. Lack G. Epidemiologic Risks for Food Allergy. *J Allergy Clin Immunol*. 2008;121(6):1331–6. doi: 10.1016/j.jaci.2008.04.032. PubMed PMID: 18539191.
16. De Benedetto A, Kubo A, Beck LA. Skin Barrier Disruption: A Requirement for Allergen Sensitization? *Journal of Investigative Dermatology*. 2012;132(3):949–63. doi: 10.1038/jid.2011.435.
17. Solensky R, Phillips EJ. Drug Allergy. In: Adkinson NF, Bochner BS, Burks W, editors. Middleton's Allergy: Principles and Practice. 9 ed. Philadelphia: Elsevier; 2022. p. 1261–82.e1.
18. Mallal S, Phillips E. The Immune Attention Economy: Keystone Epitope Theory and the Strategic Allocation between Tolerance, Equilibrium, and Sterilization. *Zenodo*. 2026. doi: 10.5281/zenodo.18520553.
19. Gibson A, Ram R, Gangula R, Li Y, Mukherjee E, Palubinsky AM, Campbell CN, Thorne M, Konvinse KC, Choshi P, Deshpande P, Pedretti S, Fear MW, Wood FM, O'Neil RT, Wanjalla CN, Kalams SA, Gaudieri S, Lehloenya RJ, Bailin SS, Chopra A, Trubiano JA, AUS-SCAR Consortium, Peter JG,

AFRiSCAR Consortium, Mallal SA, Phillips EJ. Multiomic Single-Cell Sequencing Defines Tissue-Specific Responses in Stevens-Johnson Syndrome and Toxic Epidermal Necrolysis. *Nat Commun*. 2024;15(1):8722. doi: 10.1038/s41467-024-52990-3. PubMed PMID: 39379371; PMCID: PMC11461852.

20. Karachaliou M, de Sanjose S, Roumeliotaki T, Margetaki K, Vafeiadi M, Waterboer T, Chatzi L, Kogevinas M. Heterogeneous Associations of Polyomaviruses and Herpesviruses with Allergy-Related Phenotypes in Childhood. *Ann Allergy Asthma Immunol*. 2021;127(2):191–9.e3. doi: 10.1016/j.anai.2021.04.019. PubMed PMID: 33895421; PMCID: PMC8801162.
21. Jansen MA, van den Heuvel D, van der Zwet KV, Jaddoe VW, Hofman A, Escher JC, Fraaij PL, Hooijkaas H, van Zelm MC, Moll HA. Herpesvirus Infections and Transglutaminase Type 2 Antibody Positivity in Childhood: The Generation R Study. *J Pediatr Gastroenterol Nutr*. 2016;63(4):423–30. doi: 10.1097/mpg.0000000000001163. PubMed PMID: 26881413.
22. Stein MM, Hrusch CL, Gozdz J, Igartua C, Pivniouk V, Murray SE, Ledford JG, Marques Dos Santos M, Anderson RL, Metwali N, Neilson JW, Maier RM, Gilbert JA, Holbreich M, Thorne PS, Martinez FD, von Mutius E, Vercelli D, Ober C, Sperling AI. Innate Immunity and Asthma Risk in Amish and Hutterite Farm Children. N Engl J Med. 2016 Aug 4;375(5):411-421. doi: 10.1056/NEJMoa1508749. PMID: 27518660; PMCID: PMC5137793.
23. Holbreich M, Genuneit J, Weber J, Braun-Fahrlander C, Waser M, von Mutius E. Amish Children Living in Northern Indiana Have a Very Low Prevalence of Allergic Sensitization. *J Allergy Clin Immunol*. 2012;129(6):1671–3. doi: 10.1016/j.jaci.2012.03.016. PubMed PMID: 22513133.
24. Ascherio A, Munger KL. Environmental risk factors for multiple sclerosis. Part I: the role of infection. *Ann Neurol*. 2007 Apr;61(4):288-99. doi: 10.1002/ana.21117. PMID: 17444504.
25. Ascherio A, Munger KL, Lennette ET, Spiegelman D, Hernan MA, Olek MJ, Hankinson SE, Hunter DJ. Epstein-Barr Virus Antibodies and Risk of Multiple Sclerosis: A Prospective Study. *JAMA*. 2001;286(24):3083–8. doi: 10.1001/jama.286.24.3083. PubMed PMID: 11754673.
26. Gaglione SA, Mukkamala RS, Krishna C, Smith BE, Wadsworth MH, 2nd, Jelinsky SA, Perez CR, Schmidt-Hong L, Katz EL, Gellatly KJ, Ali LR, Shen J, Holec PV, Zhao QH, Chan AO, Xu EJK, Kravarik KM, Guzova JA, Dobson CS, Singh H, Garber M, Dougan M, Dougan SK, Harris JE, Winkler A, Birnbaum ME. Scalable TCR Synthesis and Screening Enable Antigen Reactivity Mapping in Vitiligo. *Immunity*. 2026;59(2):477–93 e9. doi: 10.1016/j.immuni.2026.01.001. PubMed PMID: 41610844; PMCID: PMC12885239.
27. Cardone M, Garcia K, Tilahun ME, Boyd LF, Gebreyohannes S, Yano M, Roderiquez G, Akue AD, Juengst L, Mattson E, Ananthula S, Natarajan K, Puig M, Margulies DH, Norcross MA. A Transgenic Mouse Model for HLA-B*57:01-Linked Abacavir Drug Tolerance and Reactivity. *J Clin Invest*. 2018;128(7):2819–32. doi: 10.1172/JCI99321. PubMed PMID: 29782330; PMCID: PMC6025983.
